# Potential Therapeutic Effects of Gut Hormones, Ghrelin and Obestatin in Oral Mucositis

**DOI:** 10.3390/ijms20071534

**Published:** 2019-03-27

**Authors:** Agnieszka Stempniewicz, Piotr Ceranowicz, Zygmunt Warzecha

**Affiliations:** Department of Physiology, Faculty of Medicine, Jagiellonian University Medical College, Grzegórzecka 16 St., 31-531 Krakow, Poland; agnieszka.stempniewicz@doctoral.uj.edu.pl (A.S.); mpwarzec@cyf-kr.edu.pl (Z.W.)

**Keywords:** gut hormone, ghrelin, obestatin, oral mucositis, chemotheraphy, radiotherapy, cancer

## Abstract

Chemotherapy and/or head and neck radiotherapy are frequently associated with oral mucositis. Oral pain, odynophagia and dysphagia, opioid use, weight loss, dehydration, systemic infection, hospitalization and introduction of a feeding tube should be mentioned as the main determinated effect of oral mucositis. Oral mucositis leads to a decreased quality of life and an increase in treatment costs. Moreover, oral mucositis is a life-threatening disease. In addition to its own direct life-threatening consequences, it can also lead to a reduced survival due to the discontinuation or dose reduction of anti-neoplasm therapy. There are numerous strategies for the prevention or treatment of oral mucositis; however, their effectiveness is limited and does not correspond to expectations. This review is focused on the ghrelin and obestatin as potentially useful candidates for the prevention and treatment of chemo- or/and radiotherapy-induced oral mucositis.

## 1. Physiological Mechanisms of Maintaining the Integrity of Oral Mucosa

### 1.1. Dynamics of Cell Renewal of Oral Mucosa

Oral mucosa is constantly exposed to mechanical, chemical and thermal trauma, as well as to infectious agents. Even in physiological conditions, during the chewing process, minor traumas occur and inflammatory infiltration is frequently observed in the histological slides of the gum [1]. Epithelial cells and saliva play a key role among the defense mechanisms limiting the development of oral mucosa damage and accelerating its regeneration. Oral mucosa is the mucous membrane lining the walls of the oral cavity and separating the light of the oral cavity from the structures of the oral cavity walls located deeper. The oral mucosa consists of two basic layers: the stratified squamous epithelium and an underlying connective tissue called the lamina propria. In order to maintain the integrity of oral mucosa, it is crucial to provide a dynamic balance between the cell loss and renewal in the epithelial layer. In the basal layer of the epithelium, cells are subject to mitotic divisions; then, the newly formed cells pass through successive layers of cells towards the surface of the mucosa undergoing differentiation and maturation along the way. Once they reach the superficial layer of the epithelium, they are exfoliated into the lumen of the oral cavity. Physiologically, the time of cell renewal of the oral mucosa is short, depends on its localization and is about 4–8 days [2]. The state of the dynamic balance between cell renewal and cell loss is based on negative feedback. Under normal conditions, an increase in cell loss leads to an increase in cell renewal, while a decrease in cell loss leads to the inhibition of cell division. This mechanism plays a key role in maintaining the integrity of the oral mucosa and its rapid regeneration. In pathology, a reduction in the cellular renewal of the oral mucosa may be observed, and it may lead to the atrophy, increased susceptibility to damage and infection, and finally to the formation of ulcers.

Moreover, oral mucosa may be a source of anti-inflammatory cytokines and growth factors, reducing the development of oral mucositis and accelerating the regeneration. On the other hand, oral epithelial cells can also secrete pro-inflammatory cytokines in the presence of pro-inflammatory factors, such as Tumor Necrosis Factor-α (TNF-α) or Pathogen-Associated Molecular Patterns (PAMPs) [3,4,5,6,7].

### 1.2. Protective and Healing Effect of Saliva

Saliva is a fluid present in the oral cavity and produced by salivary glands. Saliva contributes to the digestion of food and plays an essential role in maintaining oral hygiene. The presence of mucous prevents the mechanical trauma of oral mucosa and aids in bolus formation. The protective function of saliva is related to the presence of ingredients with digestive and anti-infective effects [8]. Numerous experimental and clinical observations indicate that a reduction in saliva production leads to an increased susceptibility to oral mucosa damage and inflammation [9], delays the healing of oral wounds [9,10,11] and increases the risk of tooth loss [12,13]. Patients with a low production of saliva are much more at risk for the development of oral mucositis [2]. In addition, chemotherapy and radiotherapy lead to a significant reduction in salivary secretion [2,14,15,16].

The important role in the protective and healing effect of saliva in injures is played by biologically active peptides called growth factors. Epidermal Growth Factor (EGF), Nerve Growth Factor (NGF) and Fibroblast Growth Factors (FGFs) seem to play an especially essential role in this respect.

EGF was originally isolated from the submandibular glands of the mice [17]; later it was found that its structure is consistent with the structure of the previously discovered urogastrone [18]. Urogastrone was originally found in human and canine urine [19]. At present, both compounds are called EGF. Apart from salivary glands, EGF was also found in numerous other organs such as the duodenum, pancreas and kidney and in several body fluids such as saliva, milk, urine, gastric juice, duodenal juice and pancreatic juice [20,21,22]. Several animal and human studies have shown that EGF stimulates the proliferation, differentiation and maturation of the cells within the gastrointestinal tract [23]. EGF prevents the mucous membrane from damage by stimulating cell proliferation [24], as well as by accelerating lesion healing [25,26]. Moreover, EGF has been found to be involved in the gastric ulcer healing effect of Growth Hormone-Releasing Hormone (GH-RH) [27], as well as exhibits an inhibitory effect on gastric acid secretion in isolated gastric glands [28]. In addition, endogenous EGF seems to play an important role in the therapeutic effect of colloidal bismuth subcitrate (De-Nol) [29] and sucralfat [30] in gastric ulcers.

The administration of exogenous EGF inhibits the development of experimental acute pancreatitis in rats [31] and accelerates the recovery in different models of acute pancreatitis [32,33]. Also, endogenous EGF seems to be involved in the protection and healing of the pancreas in acute pancreatitis. Konturek et al. found an increased expression of mRNA for EGF in the pancreas during acute pancreatitis [34]. Previous studies have also shown that EGF plays an important role in maintaining the integrity of the oral mucosa. During studies on rats, Morris-Wiman et al. have found that the removal of the salivary glands results in the atrophy of the taste buds within the fungiform papillae on the tongue surface in these animals, whereas the administration of exogenous EGF reversed these changes [35]. Similar effects were found in other animals, such as mice and rabbits [10,36]. In humans, the decrease in salivary EGF level was found in patients with oral inflammation such as stomatitis aphtosa and peritonsillar abscess [37]. A low salivary EGF level is also observed in patients with Sjögren syndrome, and the degree of EGF drop in saliva exhibits a significant correlation with the deterioration of their life quality [38]. In addition, low salivary EGF levels were found in patients receiving radiotherapy to the head and neck [39,40]. On the other hand, a higher EGF level in saliva is associated with less severe oral mucosa damage due to radiation therapy [40]. These findings suggest that decreasing the salivary EGF levels reduces the capacity of the oral mucosa to maintain its integrity and limits its ability to heal after injury. Moreover, there are clinical studies showing that the oral administration of recombinant human EGF exhibits a therapeutic effect in oral mucositis in patients undergoing radiotherapy, with or without chemotherapy, for head and neck cancer [41].

NGF, a member of the neurotrophin family, is typically synthesized as a high molecular weight complex (130 kDa) consisting of β-NGF and two subunits α and γ. Subunits α and γ belong to the kallikrein family of serine proteases. β-NGF is a large precursor of NGF (pro-NGF) and is processed by α and NGF subunits to generate functional NGF [42]. NGF plays an essential role in the proliferation, growth and survival of neurons during organ development, as well as in the maintenance of neurons integrity in later periods of living [43]. In the absence of NGF, neurons undergo apoptosis [44], and this observation is in agreement with the findings that NGF exhibits a protective and antiapoptotic effect on spinal cord neurons in sciatic nerve-injured rats [45].

In addition to its effects on the nervous system, NGF has been shown to accelerate the healing of skin wounds and corneal ulcers [46,47,48]. NGF is extensively expressed in the oral cavity and produced in the animal and human salivary glands. Intercalated, striated and collecting ducts in all major and minor salivary glands exhibit a strong expression of pro-NGF but only a weak expression of mature NGF [49]. Apart from salivary glands, the pro-NGF staining is observed in all the epithelial layers of oral mucosa, whereas the expression of mature NGF was found in the granular and upper spinous cell layers [50]. Also, leukocytes and fibroblast present in oral mucosa exhibit the presence of pro-NGF and mature NGF.

There are two classes of NGF receptors, the high-affinity receptor: Tropomyosin receptor kinase A (TrkA) and the Low–affinity Nerve Growth Factor Receptor (LNGFR) also known as the p75neutrophin receptor. The activation of TrkA is essential for inducing cell survival and differentiation caused by NGF. Activating LNGFR may lead a cell to die by apoptosis; however, this effect may be counteracted by the activation of TrkA with antiapoptotic effects. Previous studies have shown that NGF receptors are expressed in the soft tissues of the oral cavity [50,51]. The data presented above indicate the potential role of NGF in oral wound healing and suggest that research in this area may lead to new therapeutic concepts [52]. On the other hand, NGF concentration in the saliva was found to be significantly and persistently increased in patients with burning mouth syndrome [53]. However it is not clear whether this is a cause, or result of burning mouth syndrome or sign of protective mechanisms activated in this syndrome.

Fibroblast Growth Factor-2 (FGF-2) known as also basic Fibroblast Growth Factor (bFGF) is a member of the Fibroblast Growth Factors Family (FGFs) [54]. Fibroblast growth factors are involved in a wide variety of processes during embryonic period and later in life. During embryonic development, FGFs play a role in regulating cell proliferation, migration and differentiation. In the adult organism, FGFs play a role in maintaining homeostasis; they regulate and are involved in tissue repair and response to damage. Their mechanism of action is related to the activation of numerous signal transduction cascades leading to the stimulation of cell growth by promoting cell cycle progression and by inhibiting pathways of cell death. For this reason, the loss of regulation at any stage of the signal transduction cascades of FGFs may promote cell growth beyond control, leading to neoplastic growth [55]. The presence of FGF-2 was found in human saliva [56], and physiologically, its concentration is age-dependent, the highest level of FGF-2 is observed in young people, while the lowest level occurs in old ones [57].

The irradiation of the head region leads to the severe damage of salivary glands and a decrease in the saliva output in clinical [58] and experimental conditions [59]. Moreover, the irradiation of salivary glands significantly reduces the microvessel density in these glands [59]. Animal studies have shown that these deleterious effects of irradiation on the microcirculation in salivary glands and the secretion of saliva can be partly but significantly reversed by a previous adenoviral vector-mediated transfer of FGF-2 complementary DNA into submandibular salivary glands by retrograde ductal delivery [59]. These observations suggest that FGF-2 may exhibit a therapeutic effect in the prevention and healing of oral mucosa.

Contrarily, there are studies showing the elevated levels of FGF-2 in saliva and serum in recently diagnosed and untreated patients with oral squamous cell carcinoma [60,61]. These observations suggest that FGF-2 is related to carcinogenesis and may increase the risk of cancer development.

Histatins are the next group of factors present in saliva and demonstrate a potential protective and therapeutic effect in the oral cavity [62]. Histatins are 12 family members of histidine-rich proteins with copper (II)- and zinc (II)-binding motifs [63]. Histatin-1 and Histatine-3 are the primary products of two genes, His1 and His2, whereas the remaining histatins are derived from Histatin-1 or Histatin-3 by proteolysis [64]. Histatins are produced in the serous acini of the salivary gland of humans and other primates [65,66] and exhibit strong antibacterial and antifungal effects [67,68], as well as stimulate the healing of oral wounds. They play an essential role in several cellular processes that take place during wound healing in the oral cavity, including the migration and spreading of keratocytes leading to the reepithelialization [69], proliferation and migration of gingival fibroblasts [70]. Moreover, Histatins stimulate endothelial and epithelial cell adhesion and improve endothelial barrier integrity, decreasing its permeability [71].

## 2. Oral Mucositis—Meet the Enemy

Oral mucositis is defined as an inflammation of the oral mucosa, characterized by the presence of erythematous areas and severe inflammation subsequently merged with ulcerations [72]. Oral mucositis is a common side effect of chemotherapy, as well as of head and neck radiotherapy [3,73]. The prevalence and severity of oral mucositis depend on the type, duration and dose of chemotherapeutics [5]. Oral mucositis is observed in 75% patients undergoing stem cell transplantation [74,75] and in 60–100% with myeloablative chemotherapy [6,76,77]. Chemotherapy and radiotherapy used together increases the risk up to 100% [3].

**Figure 1:**
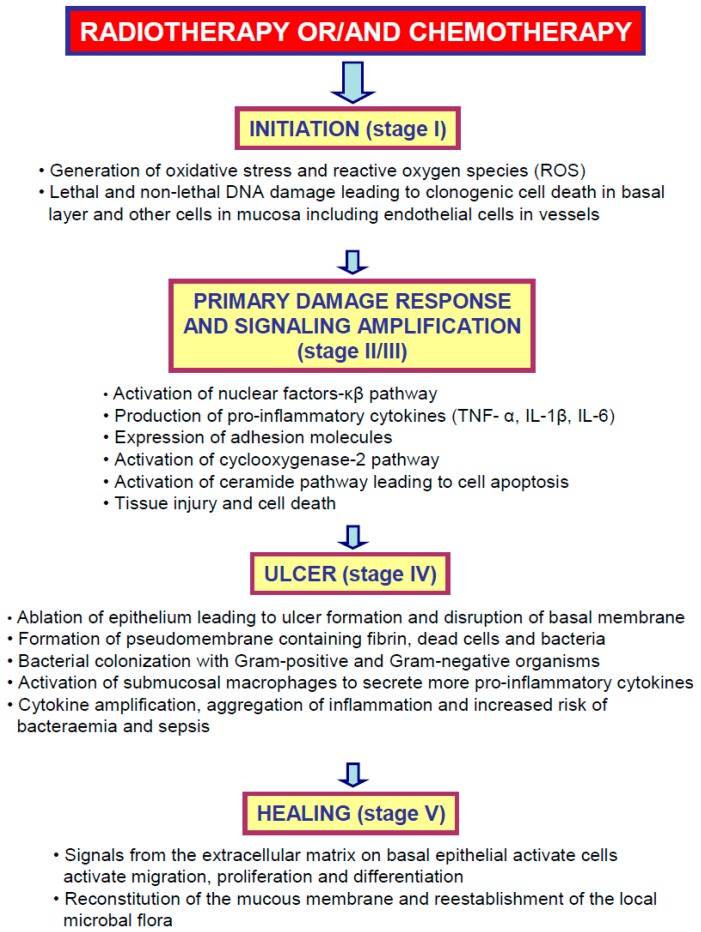
The five-stage model of the development and healing of oral mucositis: (I) initiation; (II) upregulation and generation of messenger signals; (III) signaling and amplification; (IV) ulceration with inflammation; and (V) healing.

Chemo- and radiotherapy should only target neoplastic cells; however, these therapies also exhibit an antimitotic effect on the rapidly dividing progenitor cells in different parts of the body. In the past, it was believed that oral mucositis is solely the result of the nonspecific damaging effect of chemo- or/and radiotherapy on rapidly dividing cells in the basal layer of the oral mucosa [4,6]. Currently, it was shown that the pathogenesis of oral mucositis is not so direct and simple [2,4,6]. Reactive oxygen species, pro-inflammatory pathways and metabolic bioproducts of colonizing microorganisms are believed to play a role in amplifying the tissue injury [78]. At the beginning of the 21st century, Sonis et al. [6,79] proposed a five-stage model for the development and healing of oral mucositis: (1) initiation; (2) upregulation and generation of messenger signals; (3) signaling and amplification; (4) ulceration with inflammation; and (5) healing (Figure 1).

Chemotherapy and radiotherapy cause DNA injury and reactive oxygen species generation, leading to basal cell damage in the endothelium. Affected cells release endogenous damage-associated molecular pattern molecules (DAMPs) which initiate the signaling phase with immune response including the activation of several hours [80,81]. The Nuclear Factor Kappa-B (NF-κB) is activated directly by chemotherapy or radiation as well as indirectly by receptor-bound DAMPs or ROS. The activation of NF-κB leads to the generation of pro-inflammatory cytokines, such as TNF-α, Interleukin-1β (IL-1β) and Interleukin-6 (IL-6) [82,83]. In the ulceration phase, there are deep and painful lesions of oral mucosa. Erythematous areas become visible 4–5 days after chemotherapy, and 3 to 5 days later, ulcers appear in the oral cavity [84]. Typically, ulcers have a necrotic base with an inflammatory infiltration of the margin, and they are rather not deep [6,85]. Finally, spontaneous healing of the ulcers takes place (Figure 1).

Oral mucositis leads to a significant lowering of the life quality and an increase in the cost of treatment. The presence of oral lesions is responsible for the development of local and systemic detrimental effects such as severe oral pain, difficulties with the swallowing of solid and liquid food (dysphagia), a pain or burning sensation on swallowing (odynophagia), trouble with (dysarthria), opioid use, dehydration and tube feeding. Moreover, mucosal lesions may be a gateway for opportunistic infections, systemic inflammation and, finally, death. These complications are associated with morbidity and mortality. Patient death may be a result of the above-listed direct life-threatening consequences of oral mucositis or the limitation of cancer control due to cancer treatment interruption or a reduction in the chemo- or/and radiotherapy doses [86].

The grading scale for oral mucositis plays a crucial role in the objective comparison of the toxicity of anticancer therapies and the efficiency of therapeutic methods used in the prevention and treatment. There are several independent systems for assessing the severity of oral mucositis, but the scoring scale proposed by the World Health Organization is most often used. The World Health Organization Oral Toxicity Scale measures the anatomical, symptomatic and functional signs of oral mucositis [86] and grades its severity from 0 to 4. Grade 0 means no changes; grade 1: soreness/erythema; grade 2: soreness/erythema + ulceration + patient can eat solid foods; grade 3: soreness/erythema + ulceration + patient can use a liquid diet only; and grade 4: soreness/erythema + ulceration + oral alimentation is not possible [86].

## 3. Management of Oral Mucositis

### 3.1. Prevention

During the initial visit before chemo- or radiotherapy, the patient should be assessed for the presence of risk factors for the development of oral mucositis [87]. Some risk factors, such as poor oral hygiene, preexisting oral pathology (e.g., dental caries), periodontal lesions, low body mass index, comorbid diseases (e.g., diabetes and infections), vitamin B12/folic acid deficiencies or tobacco or alcohol use, can be eliminated or reduced though preventive actions.

Among the recommended evidence-based methods for the prevention of radiotherapy and chemotherapy-induced mucositis, the following ones should be mentioned: good oral hygiene, palifermin, antioxidant agents (amifostine and GC4419) and low-level laser therapy/photobiomodulation [86].

Good oral hygiene is mandatory for minimizing the incidence and severity of mucositis in cancer patients. Shieh et al. have shown that an appropriate oral care protocol applied 1 week before radiotherapy delays the onset of mucositis and reduces the grade of this inflammation [88]. Good oral hygiene was found to reduce the bacterial load and risk of local and systemic infections [89]. The relationship between oral hygiene and the risk of complications in patients treated with radiotherapy was confirmed by the American Academy of Oral Medicine (AAOM) in 2016 [89]. AAOM recognized that preventive oral care is essential to minimize the severity of adverse effects of high-dose head and neck radiation. The oral care should be completed at least 2 weeks before the first dose of radiation [89].

Palifermin (trade name Kepivance, Swedish Orphan Biovitrum AB, Stockholm, Sweden) is a trunked recombinant human Keratinocyte Growth Factor (KGF) [90]. KGF belongs to the fibroblast growth factor family and is also known as fibroblast growth factor-7 [55]. KGF was originally isolated from the conditioned medium of a human fibroblast cell line as a growth factor specific for epithelial cells [55]. KGF and palifermin stimulate the proliferation, differentiation and survival of epithelial cells. KGF also exhibits antiapoptotic, antioxidant and anti-inflammatory effects [79]. Clinical studies showed that palifermin reduces the duration and severity of oral mucositis after intensive chemotherapy and radiotherapy for hematologic cancers [90,91], as well as decreases swallowing problems, nutrition impact symptoms and the length of stay in a hospital [91]. For this reason, palifermin was approved by the Food and Drug Administration (FDA) [92] and the European Medicines Agency (EMA) [93] for use in patients with hematologic malignances receiving myelotoxic therapy in the setting of autologous stem cell support to reduce the incidence and duration of severe oral mucositis. At the same time, the FDA warns that of the safety and efficiency of palifermin (Kepivance) has not been established in patients with non-hematologic malignances. The effect of Kepivance on the stimulation of KGF receptor-expressing, non-hematopoietic tumors in patients are unknown. Moreover, large doses of Kepivance has been shown to enhance the growth of the human epithelial tumor cell line in vitro and to increase the rate of tumor cell line growth in a human carcinoma xenograft model cell [92]. In addition, on 1 April 2016, the European Commission withdrew the marketing authorization for Kepivance (palifermin) in the European Union (EU). The withdrawal was at the request of the marketing authorization holder, Swedish Orphan Biovitrum AB (publ), which notified the European Commission of its decision to permanently discontinue the marketing of the product for commercial reasons [93].

Amifostine, as a free radical scavenger, exhibits antioxidant and cytoprotective activities [86]. Its importance as a potent radioprotector was discovered under the auspices of the Manhattan Project during the Second World War [87]. The cytoprotective effect of amifostine, apart from free-radical scavenging, involves DNA protection repair, acceleration and induction of cellular hypoxia. It protects tissues from radiotherapy and chemotherapy damage, and its effect is mainly limited to normal nonneoplastic tissues [94]. In the body, a phosphorylated aminothiol prodrug, amifostine, is converted to its active sulfhydryl metabolite by alkaline phosphatase. Alkaline phosphatase is present in large amounts in the normal endothelium. In contrast, in neoplastic tissues, alkaline phosphatase is present at a much lower level, and its activity is inhibited by the acidity of the tumor environment. Also, neoplastic tissues are characterized by a low number of vascular vessels. These mechanisms almost completely limit the possibility of forming active metabolites of amifostine in neoplastic tissues [94]. Amifostine is conventionally administered intravenously before radiotherapy or chemotherapy. In 1996, amifostine was approved by the FDA for the reduction of cumulative nephrotoxity associated with the repeated administration of cisplatin in advanced ovarian cancer [86]. Furthermore, in 1999, the FDA approved amifostine for the prevention of developing radiation-induced xerostomia in patients with head and neck cancer [86]. The clinical use of amifostine is limited due to severe toxicities such as nausea, emesis and hypotension [86,87].

The excess free radicals play an essential role in the development of oral mucositis evoked by radio- or/and chemotherapy. GC4419 is a synthetic manganese-based small molecule that mimics the antioxidant enzyme Superoxide Dismutase (SOD). GC4419, like SOD, converts a superoxide anion to a hydrogen peroxide or oxygen molecule and no other reactive oxygen species [86]. The clinical trial phase 1b/2a has shown that GC4419 may be useful in reducing the frequency and duration of severe oral mucositis in patients with locally advanced oral cavity or oropharyngeal cancer and treated with definitive or postoperative intensity modulated radiation therapy (IMRT) plus cisplatin. GC4419 was administered intravenously as a 60-min infusion, which ended less than 60 min before IMRT from Monday to Friday for 3 to 7 weeks. The side effects of GC4419 have included nausea, vomiting and paresthesies [95]. However, it should be noted that there are no data on the effect of GC4419 on the effectiveness of radiotherapy and chemotherapy in the treatment of primary patients’ disease.

There is a large number of evidences showing that photobiomodulation, formerly known as low-level laser therapy, is useful in the prevention and/or treatment of oral mucositis evoked by radiotherapy for head and neck cancer or chemotherapy [96]. The preventive use of low-level laser therapy significantly reduces the duration and severity of radiotherapy-induced oral mucositis, as well as leads to pain relief [97]. Apart from the prevention/treatment of oral mucositis, it may have an important role in supportive care for a broad range of other complications associated with radio-or/and chemotherapy [98]. For oral mucositis management, photomodulation is used usually with the following parameters: a wavelength of 633–685 or 780–830 nm; a power output of between 10 and 150mW; an energy density of 2–3 J/cm^2^ and no more than 6 J/cm^2^ on the tissue surface treated,; administered two to three times a week up to daily [98]. The most frequently used photobiomodulation devices include the Helium–Neon (HeNe) gas laser; Gallium–Arsenide (GaAs); Neodymium-doped Yttrium Aluminum Garnet (Nd:YAG); Gallium Aluminum Arsenide (GaAlAs); Indium Gallium Aluminum Phosphide (InGaAlP) diode lasers; nonthermal, non-ablative carbon dioxide lasers; Light-Emitting Diode (LED) arrays; and visible light [99].

Among the methods that were or are used to protect the oral mucosa against the development of inflammation during radio- or/and chemotherapy should also be mentioned: the oral administration of PTA (Polymixine E, Tobramycine and Amphotericin B), therapy with Granulocyte Colony-Stimulating Factor (G-CSF) or Granulocyte Macrophage Colony-Stimulating Factor (GM-CSF) and cryotherapy.

The effect of treatment with PTA on the development of oral mucositis remains unclear in patients receiving radiotherapy or chemotherapy. Clinical placebo-controlled double-blind studies, performed during last 20 years, have shown that treatment with an oral paste containing PTA [100] or the administration of active lozenges containing PTA [101] do not prevent the development of severe oral mucositis in head and neck cancer patients receiving radiotherapy. The older placebo-controlled double-blind studies have shown the same lack of an effect of PTA administration on the incidence of mucositis but suggests that the administration of PTA is able to significantly reduce the area and distribution of mucositis, the grade of dysphagia and the loss of a patient’s weight [102]. A single old paper presenting the effect of PTA on the development of oral mucositis in pediatric patients undergoing chemotherapy prior to bone marrow transplantation reported that the administration of PTA significantly reduces the severity of oral mucosa, but this effect is unlikely to produce practical benefits [103].

There is no definite and convincing evidence that the use of the Granulocyte Colony-Stimulating Factor (G-CSF) or the Granulocyte Macrophage Colony-Stimulating Factor (GM-CSF) before or during radiotherapy of head and neck cancer or chemotherapy exhibits therapeutic benefits in oral mucositis. Clinical trials on the systemic or topical application of G-CSF or GM-CSF on oral mucositis in these patients are confusing. Some especially early studies have suggested that the administration of GM-CSF [104,105,106] or G-CSF [107,108] before radiotherapy of head and neck or chemotherapy may reduce the severity and frequency of oral mucositis. However, it should be said that these studies contain numerous methodological errors, and they are double-blind placebo controlled randomized studies. Most of them were not randomized; some of them did not have a control group at all [104,107] or the control group was formed of retrospective case-matched patients [105], or an inappropriate placebo was used [108]. Some studies presented results obtained in a small group of patients consisting of 12 [105], 17 [104] or 14 [107] people.

In contrast, clinical double-blind placebo controlled randomized studies exhibit a minimal or no effect of GM-CSF [109,110,111,112] on radiotherapy- or/and chemotherapy-induce oral mucositis. In the case of G-CSF, the last double-blind placebo controlled randomized study performed in 2006 showed that the administration of G-CSF during postoperative radiotherapy for head and neck cancer exhibits a tendency to lower the rates of percutaneous endoscopic gastrostomy placement and the severity of oral mucositis, but these effects were not statistically significant [113].

Oral cryotherapy involves the cooling of the oral cavity using ice cubes, ice chips, cold water or ice cream [114]. It has been shown to be effective in the prevention of chemotherapy-induced oral mucositis in adult patients receiving fluorouracil-based chemotherapy [115] or fluorouracil combined with leucovorin [116] for solid cancer. A similar protective effect of cryotherapy on oral mucosa has been found in adult patients receiving melphalan-based chemotherapy before Hematopoietic Stem Cell Transplantation (HSCT) [117] and in patients receiving chemotherapy containing docetaxel together with cisplatin and fluorouracil for the treatment of esophageal cancer [118]. Cryotherapy reduced the incidence and severity of oral mucositis, as well as reduced the incidence of anorexia [118]. It is believed that the preventive effect of cryotherapy against the development of chemotherapy-induced oral mucositis is associated with the influence of the cold on local microcirculation. The cold causes vasoconstriction and reduces local blood flow, which in turn limits the amount of cytostatic drugs delivered to the oral mucosa and finally reduces mucosal damage [116].

### 3.2. Treatment

The treatment of oral mucositis is mainly symptomatic and should reduce the side effects of chemo- or/and radiotherapy and oral mucositis. Patients suffer, among others, from a loss of taste, xerostomia, nausea and vomiting, odynophagia or dysphagia. If patients are able to eat, a diet restriction should include the elimination of acidic, salty and spicy food, as well as alcohol [119]. In the case of severe oral mucositis, patients are not able to consume orally solid or even liquid food. Nutritional counseling and oral nutritional supplements should be used to increase dietary intake and to prevent therapy-associated malnutrition and the interruption of the anticancer therapy [119]. Moreover, a deterioration of the nutritional status results in an increase in anticancer-related toxicity and may prolong the treatment time, leading to poor clinical outcomes. If the patient cannot eat orally, enteral nutrition via nasogastric tube or percutaneous gastrostomy [119] or even total parenteral nutrition should be introduced [120]. Some reports suggest that enteral feeding is more beneficial than parenteral nutrition in patients undergoing allogeneic hematopoietic cell transplantation due to hematological malignances. Adequate enteral feeding during the early phase of transplantation course is associated with a reduced non-relapse mortality, improved survival and graft-versus-host disease-free/relapse-free survival at 5 years. Also, adequate enteral feeding is associated with a lower incidence of overall and gut acute graft-versus-host disease than parenteral nutrition [121]. To prevent dehydration and to reduce xerostomia, the patient should be provided with adequate amounts of fluids.

Pain control plays an essential role in the maintenance of life quality and oral feeding. Some authors recommend the use of anesthetics and analgesics for a reduction of the pain in oral mucositis. Most therapeutic regimens are empirical with no scientific basis. The administration of local anesthetics such as lidocaine (also known as Xylocaine or Lignocaine), bupivacaine or dyclonine leads to a transitory pain relief in oral mucositis. Various lidocaine preparations, in the form of gels, sprays or viscous solutions, are currently used for the local anesthesia of the oral cavity [86,122]. The onset of its action is approximately 1–2 min, with a relatively short duration of action lasting about 15 min and a peak efficacy occurring at 5 min [122]. On the other hand, clinical studies conducted by Mogensen et al. on patients with head and neck cancer receiving radiotherapy with or without concurrent chemotherapy have shown that treatment with bupivacaine lozenges (taken up every 2 h) plus standard oral mucositis pain treatment is more effective in pain control than treatment with topical lidocaine plus standard pain treatment [123]. Also, dyclonine seems to exhibit a stronger and longer-lasting analgesic effect on oral mucositis than lidocaine [124]. Paracetamol, nonsteroidal anti-inflammatory drugs (NSAID) and opioids are currently the most frequently used drugs to achive systemic analgesia [124,125,126], but only limited data on the most beneficial analgesic therapy are available. Between these medicines, opioids play an essential role in pain relief in patients with oral mucositis [125,126]. Morphine may be administered parenterally [125,126] or orally as mouthwashes alone [127,128] or oral morphine rinse combined with the oral intake of a morphine solution [129]. Another option is transdermal fentanyl which has been shown as an effective, convenient and well-tolerated treatment for severe mucositis pain. Treatment with transdermal fentanyl markedly improves the quality of life in patients with oral mucositis evoked by chemotherapy [130]. Moreover, experimental studies have shown that, apart from the analgesic effect, opioids stimulate the cell migration of oral epithelial cells by delta-opioid receptor activation [131]. This observation suggests that opioids may also be involved in the re-epithelialization of the oral cavity.

Radiotherapy for head and neck cancer leads to oral mucositis, the damage of the salivary glands and xerostomia. Also, chemotherapy is associated, in most cases, with the development of oral mucosistis and xerostomia [132]. For this reason, patients use a range of mouthwashes. It provides relief for patients by moisturizing the mucous membrane and reducing pain. The composition of mouthwashes depends on the manufactures-specific recipes. They may contain various compounds, including topical anesthetic, steroids, NSAID, antiseptics, antibacterial and antimycotic agents [79,86,133].

Some relief of pain in oral mucositis may be reached by bioadherent oral gels or ointments. They adhere to the surface of the oral mucosa, creating a barrier between the light of the mouth and oral lesions. This effect protects the mucosa membrane against oral irritating and the harmful effect of oral content. Gelclair^®^, Orabase^®^, Episil^®^ and MuGard^®^ are the most commonly used protectants for the oral mucosa membranes. They reduce pain in the mouth, but unlike typical mouthwashes, the effect lasts longer after a single administration. Gelclair**^®^** is produced by Helsinn Healthcare SA (Lugano, Switzerland) and contains as active ingredients, among others, maltodextrin, propylene glycol, polyvinylpyrrolidone (povidone), sodium hyaluronate, hydroxyethylcellulose and glycyrrhetinic acid [134]. Orabase Protective Paste**^®^**, produced by ConvaTec, contains gelatin, pectin and carboxymethylcellulose sodium in plasticized hydrocarbon gel (polythene and liquid paraffin). Episil**^®^** (Camurus AB, Lund, Sweden) is composed of glycerol dioleate, phosphatidylcholine (soy lecithin), ethanol, propylene glycol, polysorbate 80 and peppermint oil. MuGard**^®^** contains purified water, glycerin, benzyl alcohol, sodium saccharin, Carbomer Homopolymer A, potassium hydroxide, citric acid, polysorbate 60 and phosphoric acid.

The role of Gelclair in oral mucositis was tested in some clinical studies. Studies performed by Barber et al. have shown that Gelcleir does not produce better therapeutic effects than standard therapy with surcralfate plus Mucaine in relieving oral pain in radiotherapy-induced oral mucositis. There was also no reduction in pain on speaking and no improvement in the ability to eat and drink [135]. Partly in line with the above observations are results obtained by Vokurka et al. [136], who found that the administration of Gelclair^®^ does not significantly improve oral food intake or oral pain control in comparison to the effects of treatment with a rinsing solution containing chlorhexidine, benzydamine and salvia (control group) in patients with oral mucositis after allogeneic stem cell transplantation. On the other hand, the duration of pain relief after a single dose of Gelclairx^®^ was statistically longer than after rinsing the mouth with the control solution. Moreover, Gelclair^®^ significantly reduced the colonization of the oral cavity by *Enterococcus faecalis* and *Candida spp*. [136]. Also, studies performed by Rasero suggest that Gelclair^®^ does not significantly reduce the severity of oral mucositis and oral pain in comparison to standard mouthwash in patients undergoing hematopoietic stem-cell transplantation [137].

The local analgesic effect of Episil^®^ in cancer patients with oral mucositis has been reported by Cheng et al. [138] who have found that Episil exhibits an efficacious local analgesic effect in cancer patients with oral mucositis following chemotherapy and/or radiotherapy [138]. A similar effect was observed by Allison et al. in the case of MuGard^®^ [139]; this multi-institutional, randomized, double-blind, placebo-controlled trial has shown that MuGard^®^ effectively mitigates oral mucositis symptoms such as oral soreness and World Heath Organisation (WHO) oral muscositis score on the last day of radiation therapy in patients treated with chemoradiation therapy for head and neck cancer [139].

Some clinical studies with a low number of participants suggest that the systemic and especially topical administration of vitamin E reduces oral mucositis induced by cancer chemo/radiotherapy [86,140]. Some beneficial effects in oral mucositis have been also observed after treatment with vitamin A [86,140].

Currently, natural organic agents are under investigation to determine their protective and therapeutic activity in the management or oral mucositis in cancer patients [141]. Some of them have shown a certain therapeutic improvement in a comparison to the control group. For example, Motallebnejad et al. have found that the oral administration of honey before and after each series of radiation significantly reduces oral mucositis in patients with head and neck cancer receiving radiotherapy [142]. Some protective or therapeutic effects have been also observed after the administration of Aloe vera juice [143], curcumin mouthwash [144,145], olive life extract [146], propolis [147,148] or chamomile [149,150]. In light of the presented results, natural agents seem to be promising alternatives in the treatment of oral mucositis evoked by chemo- or/and radiotherapy. However, it should be stated that the abovementioned studies are characterized by a low number of observations and a large degree of freedom in conducting tests and evaluating their results. Therefore, to confirm the correctness of the obtained results, double-blind, randomized, multicenter, placebo-controlled studies with an appropriate number of observations are required.

Melatonin is the next new strategy for prevention and treatment of oral mucositis evoked by chemo- or/and radiotherapy. Melatonin is synthesized in the pineal gland from the amino acid tryptophan. The synthesis of melatonin occurs also, among others, in the oral mucosa and salivary glands [151]. Melatonin exhibits anti-inflammatory and anti-oxidative effects; it increases the expression and activity of antioxidant enzymes such as superoxide dismutase, catalase, glutathione peroxidase, glutathione reductase and γ-glutamyl cysteine synthase. For this reason, melatonin inhibits the development of oxidative stress. Other protective effects of melatonin involve preventing mitochondrial dysfunction and DNA degradation and inhibiting the inflammatory response, apoptosis and the NF-κB pathway. Moreover, melatonin exhibits a strong radioprotective effect. It reduces radiation-induced DNA damage, oxidative stress in mitochondria, lipid peroxidation, inflammatory response and apoptosis [152]. On the other hand, melatonin and its metabolite enhance the sensitivity of cancer cells to anticancer drug [153]. Clinical studies indicate that the administration of melatonin during chemotherapy significantly increases the survival rate and objective tumor regression [154] and reduces some chemotherapy-induced side effects, such as myelosuppression, neurotoxity thrombocytopenia, cardiotoxocity and oral mucositis [154,155]. Moreover, recent clinical studies have shown that the concomitant administration of melatonin delays the onset of oral mucositis and reduces the amount of morphine used for pain treatment in head and neck cancer patients receiving radio- and chemotherapy [156].

The effectiveness of current therapeutic methods is limited. They are not able to prevent the development of oral mucositis in cancer patients treated with radio- and chemotherapy. On the other hand, some of them can delay the development of this inflammation, reduce its severity and duration and improve the quality of patient’s life. However, it is not in line with patients’ expectations. Efforts should be made to find new methods for the prevention and treatment of oral mucositis in patients at risk of developing this inflammation. Experimental studies suggest that two hormones, ghrelin and obestatin, may be useful in solving this problem.

## 4. Ghrelin and Its Main Physiological Effects

In 1999, ghrelin was discovered in human and rat stomach by Kojima et al. [157]. Ghrelin is a 28-amino acid peptide, and it has been found to be a natural ligand for growth hormone secretagogue receptors (GHS-R). There are two types of the growth hormone secretagogue receptors: GHS-R1a and GHS-R1b. Ghrelin is a ligand for both receptors, but only GHS-R1a is biologically active [158,159]. The physiological role of GHS-R1b is unknown, but it is possible that it protects cells that have receptors of this type against an excessive stimulation by ghrelin.

Currently, GHS-R1a is called the ghrelin receptor to follow the convention of naming receptors after the endogenous agonist [160]. The ghrelin receptor is a G-protein-coupled receptor and signals via a G_q/11_ α-subunit that results in the activation of phospholipase C and the production of Inositol Triphosphate (IP_3_) and that releases Ca^2+^ from the endoplasmic reticulum [160,161]. The human ghrelin gene is located on chromosome 3 at locus 3p25–26 [162]. The ghrelin gene undergoes transcription and translation into a 117-amino acid preproghrelin that is the precursor for ghrelin and obestatin [159]. Moreover, Seim et al. have demonstrated the presence of ghrelin gene-derived mRNA transcripts that do not encode ghrelin but, instead, may encode the C-terminal region of full-length preproghrelin (C-ghrelin) and a transcript encoding only obestatin. In addition, they have also found several natural antisense transcripts, termed ghrelinOS (ghrelin opposite strand) transcripts. The presence of these sense and antisense alternative transcripts suggests that ghrelin gene-derived peptides may be also produced independently of preproghrelin [163].

In the circulation, ghrelin exists in two different forms, as acylated ghrelin (acyl ghrelin) and non-acylated ghrelin (des-acyl ghrelin) [157]. Acyl ghrelin is an active form of ghrelin. The acylation of ghrelin is necessary for combining ghrelin with its receptor. Ghrelin is acylated mainly by octanoic or decanoic acid on the N-terminus of serine-3 amino acid [159]. The acylation of ghrelin is carried out by an enzyme called ghrelin-O-acyltransferase (GOAT) [164]. The half-life of acyl ghrelin is only about 11 min, whereas the half-life of desacyl ghrelin is 29 min [165]. Ghrelin is predominantly secreted in the stomach. However, it was also found in other organs such as the intestine, pancreas, kidney, pituitary gland and hypothalamus [157,166,167]. The ghrelin receptor occurs mainly in the pituitary gland and hypothalamus. Its presence was also found, but in much smaller quantities, in other organs such as the thyroid gland, pancreas, spleen, myocardium, adrenal gland, gonad, heart, lung and cells of the immunological system [166,168,169].

Studies in humans and on animals have shown that ghrelin strongly and dose dependently stimulates the release of growth hormone from the anterior pituitary [157]. It is mainly the result of the direct action of ghrelin on the ghrelin receptors present on pituitary somatotropes. Moreover, ghrelin also stimulates the liberation of growth hormone via an indirect pathway. Ghrelin acting on neurons expressing growth hormone-releasing hormone (GH-RH) in the hypothalamus leads to the release of GH-RH by these neurons. Then, GH-RH via pituitary microcirculation reaches somatotropes in the anterior part of the pituitary and stimulates them to release the growth hormone [170]. In addition to growth hormone, ghrelin also promotes the release of adrenocorticotropic hormone (ACTH), cortisol and prolactin [171,172].

Numerous animal and human studies have evaluated the effect of ghrelin on food intake, body weight, energy expenditure and glucose homeostasis [173]. Ghrelin stimulates appetite and fat deposition in mature rats [174,175,176,177,178] and humans [179,180,181,182]. This is associated with body mass gain and a decrease in fat utilization [173,183]. Plasma ghrelin level is negatively correlated with body mass index and energy balance. Starvation, anorexia nervosa and cachexia increase the plasma concentration of ghrelin [184,185,186,187], whereas obesity and food intake reduce the plasma concentration of ghrelin [184,186,188]. The grade of plasma ghrelin suppression by food depends on the nutrient type. The strongest reduction in the ghrelin concentration is observed after protein ingestion; a smaller effect is observed after the ingestion of carbohydrates, and the smallest one is observed after the intake of lipids [189].

The food intake-promoting (orexigenic) effect of ghrelin involves central and peripheral mechanisms. The hypothalamic arcuate nucleus mediates the anorectic effects of leptin and orexogenix effect ghrelin, and the neurons of the arcuate nucleus exhibit a high density of ghrelin receptors [190]. The leptin- and ghrelin-responsive network involves the hypothalamic neuropeptide Y/α-melanocyte stimulating hormone (NPY/α-MSH) system. Ghrelin exerts excitatory effects on the neurons present in the ventromedial part of arcuate nucleus and expresses NPY, agouti-related protein (AgRP) and orexin [190,191,192]. In contrast, a high number of neurons present in the ventrolateral part of the arcuate nucleus (expressing pro-opiomelanocortin (POMC) and synthesizing the anorectic peptide α-MSH) is inhibited in response to ghrelin [190]. These results indicate that circulating ghrelin may directly oppose the effects of leptin by the activation of NPY-neurons in the ventromedial part of arcuate nucleus and indirectly by the inhibition of POMC neurons in the ventrolateral part of arcuate nucleus in the hypothalamus [190].

Ghrelin and cholecystokinin (CCK) are gastrointestinal hormones regulating feeding. The first step in the integration of hunger and satiety signals is related to the peripheral level of ghrelin and CCK and takes place at the level of the vagus nerve [193,194]. Date et al. have found that a blockade of the vagal afferent pathway abolishes ghrelin-induced feeding, suggesting that the vagal afferent pathway conveys ghrelin orexigenic signals to the nucleus tractus solitarius (NTS) [194]. Moreover, Date et al. have found that the orexigenic effect of peripherally administered ghrelin involves the noradrenergic system in the central control of feeding behavior [194].

Previous studies have also shown that the regulation of ghrelin secretion by energy balance, as well as the influence of ghrelin on food intake and weight gain is age-dependent and imperfect at an early stage of life. For example, the study performed on prepubertal children has shown that ghrelin secretion in childhood is refractory to the inhibitory effect of feeding [195,196]. Similar atypical relationships between the level of ghrelin and nutrition are also observed in young immature animals. The intraventricular administration of ghrelin does not stimulate but inhibits food intake in freshly hatched chicks [197]. Also, the peripheral administration of ghrelin in suckling or weaned rats reduces body weight gain and daily food intake and inhibits the growth and functional maturation of the pancreas [198] and stomach [177].

Among the physiological functions of ghrelin should also be mentioned the stimulation of gastric motility and gastric emptying [199,200]. The influence of ghrelin on the exocrine secretory activity in the stomach is uncertain. Early studies have suggested that the intravenous [199] and intraventricular [201] administration of ghrelin in urethane-anesthetized rats stimulates gastric acid secretion. On the other hand, studies performed in conscious rats with chronic gastric fistulas or a ligation of the pylorus have suggested that ghrelin did not affect the exocrine secretory activity of the stomach [200], whereas the third group of studies reported that the central administration of ghrelin inhibits gastric acid secretion in conscious rats [202].

The administration of ghrelin inhibits pancreatic exocrine pancreatic secretion [203]. However, the effect of ghrelin on pancreatic endocrine secretion has been controversial. Early studies have reported that ghrelin stimulates insulin secretion [204,205], whereas other reports have showed an inhibitory effect of ghrelin on insulin release by pancreatic β-cells [206,207]. New studies have solved this problem, and currently, it is generally accepted that ghrelin, acting directly on islet β cells, inhibits glucose-dependent insulin secretion [208].

## 5. Protective, Therapeutic and Anti-Inflammatory Effects of Ghrelin

**Figure 2:**
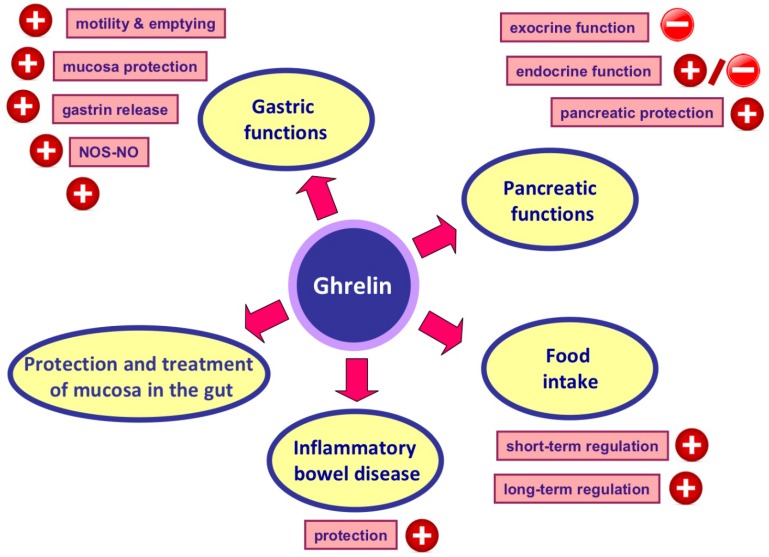
A schematic representation of the ghrelin effects in the gut. Plus means increases stimulates; minus meas inhibits.

Numerous animal studies have demonstrated that exogenous ghrelin exhibits a protective effect in several organs such as the heart [209], kidney [210] and brain [211] against ischemic injury, as well as reduces the severity of sepsis-induced lung injury and mortality [212]. Moreover, ghrelin exhibits therapeutic effects in different experimental models of inflammation or tissue damage, among others, in autoimmune encephalomyelitis [213], nerve regeneration [214] and wounds evoked by a combination of radiation plus burns [215]. In the gut, protective and therapeutic effect of ghrelin administration has been shown, among others, in the stomach, duodenum and colon (Figure 2). Pretreatment with ghrelin protects gastric mucosa against damage evoked by ethanol [216], stress [217] or alendronate [218]. A beneficial effect of ghrelin administration has been also shown in the treatment of experimental ulcers in different organs. Treatment with ghrelin accelerates the healing of gastric, duodenal and oral ulcers evoked by acetic acid, ethanol or cysteamine [219,220,221]. The healing-promoting effect of ghrelin in these ulcers has been associated with the recovery of adequate mucosal blood flow and an improvement of the mucosal cell vitality and proliferation, an increase in antioxidant defense, as well as a reduction in the mucosal oxidative stress and inflammatory response. Moreover, Wu et al. [222] have shown that the level of endogenous ghrelin is significantly reduced after an ischemia/reperfusion (I/R)-induced intestinal injury. The administration of exogenous ghrelin inhibits pro-inflammatory cytokine release, reduces neutrophil infiltration in the intestine and lung, ameliorates intestinal barrier dysfunction, attenuates intestinal and pulmonary injury and improves the survival of rats after ischemia/reperfusion-induced intestinal injury [222].

Accelerating the healing in gastric and duodenal ulcers is related to an improvement of the gastri and duodenal mucosal blood flow, an increase in mucosal cell proliferation and antioxidant defense, as well as a reduction in mucosal oxidative stress and inflammatory response. Treatment with ghrelin increased the serum level of GH and IGF-1 [157,219].

Ghrelin exhibits a protective and therapeutic effect also in the colon. Ghrelin reduces the severity of colitis evoked by trinitrobenzene sulfonic acid [223,224]. The administration of ghrelin has been also shown to inhibit the development of colitis evoked by acetic acid [225] or dextran sodium sulfate (DSS) [226,227]. In a mouse model of colitis evoked by DSS, Cheng et al. have found that ghrelin inhibits the development of colitis and prevents the breakdown of intestinal barrier function, and these effects seem to be related to the ghrelin-induced inhibition of NF-κB activation [228].

Next, experimental studies have shown that ghrelin administered intraperitoneally after the induction of colitis evoked by acetic acid leads to the acceleration of healing of colitis [229]. This effect has been observed in macroscopic and microscopic examinations. Macroscopic examination has shown a reduction of the area of colonic damage, whereas microscopic examination has revealed that ghrelin administration reduces the grade of colonic damage and inflammatory infiltration, decreases the depth of lesions and prevents the development of fibrosis [229]. These effects were associated with an improvement of the colonic blood flow and DNA synthesis in colonic mucosa. This last effect indicates that the administration of ghrelin improves mucosal cell vitality and proliferation. These effects play an essential role in the regeneration of damages tissues. Moreover, ghrelin has shown a strong anti-inflammatory effect in acetic acid-induced colitis. The colitis-evoked increase in the mucosal presence of proinflammatory IL-1β and TNF-α was significantly reduced. Also, the mucosal activity of myeloperoxidase, a biochemical marker of leukocyte inflammatory infiltration, was significantly reduced in animals with colitis and treated with ghrelin in comparison with animals with colitis and treated with a placebo [229]. The next studies were performed to examine the role of growth hormone and insulin-like growth factor-1 (IGF-1) in the therapeutic effect of ghrelin in the course of acetic acid-induced colitis [230]. The studies were performed in pituitary-intact or hypophysectomized rats. In pituitary-intact rats, ghrelin stimulated the release of growth hormone from the pituitary gland and increased the serum level of IGF-1, and these effects were associated with therapeutic effects of ghrelin in acetic acid-induced colitis [230]. Hypophysectomy increased the severity of acetic acid-induced colitis and eliminated the effect of ghrelin on growth hormone and IGF-1 secretion, as well as abolished the healing-promoting effect of ghrelin on colitis. These effects strongly suggest that the therapeutic effect of ghrelin in colitis is indirect and mediated by the release of endogenous growth hormone and IGF-1 [230].

In contrast to the above observation, in vitro studies performed by Zhao et al. suggest that ghrelin may participate, in some conditions, in the induction or aggravation of colonic inflammation [231]. The exposure of non-transformed human colonic epithelial NCM460 cells stably transfected with ghrelin receptor mRNA increased IκaBα phosphorylation and its subsequent degradation. It led to an increased NF-κB-binding activity, NF-κB p65 subunit phosphorylation and the stimulation of proinflammatory interleukin-8 (IL-8) promoter activity and IL-8 protein secretion. Moreover, Zhao et al. have reported that this effect has been markedly reduced by pharmacological inhibitors of intracellular calcium mobilization (BAPTA/AM) and that protein kinase C are involved in the activation in this proinflammatory pathway [231]. However, it should be noted that such a setting of the experiment is extremely artificial and, therefore, does not correspond to any clinical conditions.

Animal studies have also shown that the administration of ghrelin protects the liver, pancreas and remote organs against direct damage and oxidative injury evoked by either the bile duct or common pancreaticobiliary duct ligation [232]. The protective and therapeutic effect of ghrelin has been also found in the pancreas. Pretreatment with ghrelin inhibits the development of experimental acute pancreatitis evoked by cerulein [233], pancreatic ischemia with reperfusion [234] and sodium taurocholate [235]. The protective effect of ghrelin was observed as a reduction in the pancreatic damage in a histological examination, a decrease in plasma level of lipase and proinflammatory IL-1β, and an improvement of pancreatic DNA synthesis [233,234]. Moreover, as in the colon [230], the protective effect of ghrelin on the pancreas has been shown to be related to the ghrelin-induced release of endogenous growth hormone and IGF-1, as well as to the inhibition of NF-κB expression [235]. Moreover, Zhou et al. [236] have found that ghrelin attenuates the severity of acute lung injury induced by acute pancreatitis. This preventive effect of ghrelin seems to be related to a reduction of neutrophil infiltration, leading to a limitation of proinflammatory cytokines release and the inhibition of substance P (SP) expression in pulmonary tissue [236].

Ghrelin exhibits a therapeutic effect in the course of animal models of acute pancreatitis. The administration of ghrelin after the development of acute pancreatitis accelerates the recovery in acute pancreatitis induced by cerulein [237] and pancreatic ischemia with reperfusion [238]. In addition, it was demonstrated that endogenous growth hormone and IGF-1 play an essential role in the therapeutic effect of ghrelin in acute pancreatitis [239]. Furthermore, in vitro studies performed on isolated rat acinar cells or rat pancreatic acinar tumor cell line AR42J have shown that the incubation of these cells with cerulein reduces the expression of ghrelin and the ghrelin receptor at the mRNA and protein levels [240]. On the other hand, the administration of ghrelin, in animals before the isolation of acinar cells, led to an increase in the expression of ghrelin and the ghrelin receptor in these isolated cells and reversed the cerulein-induce inhibition of their expression. The same effect was observed in AR42J cells after incubation with cerulein and a ghrelin or combination thereof [240]. In addition, studies performed on rats indicate that capsaicin-sensitive sensory nerves are necessary for the protective effect of ghrelin in cerulein-induced acute pancreatitis [241].

Clinical observations have shown that acute pancreatitis affects the serum level of ghrelin. However, the character of changes remains unclear. First of all, there are large differences in the levels of ghrelin and the direction of changes in the level of ghrelin between reports. Secondly, in most cases, there is no control group without acute pancreatitis. Moreover, there are differences in the time of blood sample collection. For example, Liu et al. [242] collected blood samples from patients with acute pancreatitis, but no control group without pancreatitis was established. Blood samples were taken twice from each patient, the first collection at the time of diagnosis and the second collection one day before the release of patients from the hospital. At the day of diagnosis, the plasma level of ghrelin reached 222 pg/mL and was significantly lower than at discharge.

These observations are partially contradictory to those obtained by Daniel et al. [243]. Daniel et al. have used the study group consisting of 32 patients with alcoholic acute pancreatitis and 30 healthy controls matched to patients by age, sex and body mass index (BMI). In all cases, acute pancreatitis was classified as grade C according to Balthazar’s computed tomography score and as severe (3 points) according to Ranson criteria. The serum levels of ghrelin measured on the 1st, 3rd and 5th day of hospitalization were comparable and significantly higher than compared to the controls.

Ulger et al. [244] carried out observations on 40 patients with gallstone-related acute pancreatitis, including 8 patients with severe acute pancreatitis and no control group. Two blood samples were collected from each patient, the first one at the day of the patient admission to the hospital and the second one at patient dischrge. All samples were collected after at least 6 h of fasting. The ghrelin levels at discharge were higher than those at admission, and this effect was statistically significant in patients with mild acute pancreatitis [244].

A higher level of ghrelin on the day of discharge than admission in patients with acute pancreatitis was found by Lee et al. [245]. Moreover, at admission, the ghrelin concentration was significantly higher in patients at high risk of developing severe pancreatitis than in patients with a low risk (286 vs. 176 pg/mL). However, the ghrelin concentration did not differ significantly between these two groups after 48 h and at discharge. Unfortunately, there was no control group.

Wang et al. [246] have reported that, on the 1st day of hospitalization, the serum level of ghrelin is significantly lower in patients with acute pancreatitis as compared to the control group. Moreover, during the first day, as acute pancreatitis severity increases, the serum ghrelin concentration decreases, being lower in patients with severe acute pancreatitis than in those with mild or moderate type (*p* < 0.05). Not to mention, the serum ghrelin level becomes progressively higher during the first five days of hospitalization. The authors stated that the receiver-operating characteristic (ROC) curves demonstrating serum ghrelin level at the first day of hospitalization had some predictive value for acute pancreatitis severity. In addition, the results presented by Wang et al. show that the serum ghrelin level in patients with acute pancreatitis exhibits an upward trend, but this effect was statistically insignificant, probably due to short time of observation [246].

Only one observation regarding the level of ghrelin in acute pancreatitis has been confirmed by most authors. Almost all of them stated that the level of ghrelin during pancreatitis grows systematically and reaches the highest value at discharge or on the last day of observation according to the study design. This observation suggests that endogenous ghrelin is involved in the recovery process in the course of acute pancreatitis.

## 6. Ghrelin as a New Weapon in the Treatment of Oral Mucositis

There are numerous important finding showing the involvement of endogenous ghrelin in physiology and pathology of the oral cavity. Ghrelin is produced and released by parotid and submandibular salivary glands but not sublingual salivary glands [247,248]. Ghrelin protein is widespread in the cytoplasm of striated, intercalated and excretory ducts, as well as in serous acini of these glands [248]. Moreover, the presence and/or expression of ghrelin have been found in teeth, taste buds of the tongue, gingival epithelium, as well as fibroblasts in the lamina propria [249,250,251]. The presence of ghrelin in molars during mouse tooth development has been investigated by Liu et al., and they have found that ghrelin is initially expressed in the inner enamel epithelium and the adjacent mesenchymal cells below. After that, the persistent expression of ghrelin was observed in the ameloblasts and odontoblasts during following developmental stages. Additionally, ghrelin was present in Hertwig’s epithelial root sheath at the beginning of tooth root formation [252].

Ghrelin receptors (GHS-R1a and GHS-R1b) have been detected in oral epithelial cells [249], periodontal ligament cells and gingival fibroblasts [253]. Moreover, the gene expression of GHS-R and the production of GHS-R1 protein is increased in periodontal ligament cells and gingival fibroblasts after stimulation by pro-inflammatory IL-1β [253]. This observation suggests that ghrelin may be involved in endogenous protective mechanisms limiting local inflammation. It is additionally supported by findings obtained by Ohta et al. [249], who have found that ghrelin inhibits the production of proinflammatory IL-8 by human oral epithelial cells stimulated by TNF-α or lipopolysaccharides [249].

Ghrelin is present in saliva in similar or even higher concentrations than in plasma or serum [247,248,249,254,255], whereas the highest concentration of ghrelin occurs in gingival crevicular fluid [249].

Clinical data indicate that the ghrelin level in the gingival crevicular fluid is lower in patients with chronic periodontitis than in healthy individuals [256]. The studies performed on human periodontal cells indicate that their exposure to *Fusobacterium nucleatum*, a pathogen involved in peridontitis development, leads to an initial upregulation and the subsequent downregulation of ghrelin receptor in periodontal cells [257]. Furthermore, gingival biopsies from patients with peridontitis have shown that inflamed periodontal areas had significantly lower ghrelin receptor expression than the healthy ones. These data indicate that the expression of the functional ghrelin receptor in periodontium is modulated by periodontal bacteria. The long-term exposure of the gingiva to periodontal bacteria downregulated the functional ghrelin receptors in gingival cells, leading to the diminution of anti-inflammatory ghrelin actions, as well as, resulting in enhanced periodontal inflammation and tissue destruction [257].

Experimental studies performed on rats have shown that the intraperitoneal administration of ghrelin significantly accelerated the healing of oral ulcers and that this effect occurs in both non-sialadenectomized and sialadenectomized rats. The beneficial effect of ghrelin is associated with a reduction of mucosal IL-1β concentration and an improvement of mucosal blood flow, cell vitality and proliferation. These finding have been confirmed and extended by studies performed on animals with intact or removed pituitary glands [258]. In pituitary-intact rats, the peritoneal administration of ghrelin significantly increased serum growth hormone and IGF-1 concentration, and this effect was associated with a significant increase in the healing rate of gingival ulcers, mucosal blood flow and DNA synthesis, as well as a reduction in local inflammation. In hypophysectomized rats, serum growth hormone was below the detection limit; the serum concentration of IGF-1 was reduced by 90%. On the other hand, hypohysectomy was without any significant effect on the healing rate of gingival ulcers, DNA synthesis or concentration of pro-inflammatory IL-1β in gingival mucosa. In hypophysectomized rats, the intraperitoneal administration of ghrelin was without any significant effect on the serum level of growth hormone and IGF-1, the healing rate of gingival ulcers, the mucosal blood flow, DNA synthesis or the concentration of IL-1β in gingival mucosa [258].

The observations presented above strongly suggest that treatment with ghrelin could be useful in the prevention and/or therapy of oral mucositis. The administration of ghrelin in oral mucositis should be recognized as a supplementation in the ghrelin deficiency and the restoration of physiological condition. This concept is supported by the following facts:

The concentration of ghrelin in saliva is similar or even higher than in plasma [247,248,249,254,255]; radiotherapy for head and neck cancer causes salivary glands damage and reduces saliva secretion [14,15,16]; chemotherapy is also able to reduce saliva secretion [259,260,261,262]; and patients with decreased saliva secretion are prone to the development of oral mucositis [2]. It is most likely that all the factors listed above reduce ghrelin level in the oral cavity.

In addition to the potential protective and therapeutic effects of ghrelin in oral mucositis, there is one more benefit arising from its use. Oral mucositis, chemotherapy, radiotherapy and cancer, the primary cause of chemo- and radiotherapy, reduce food intake, leading to weight loss. The administration of ghrelin increases food intake and appears to be a promising therapeutic option for cancer cachexia [263].

On the other hand, it is necessary to determine what form of ghrelin should be used in oral mucositis, as well as, the question about of the route of it administration: As a peptide or synthetic non-peptide analog? Parenterally or orally? Only new studies could answer to these questions.

## 7. Obestatin and Its Protective and Therapeutic Effects

Obestatin was discovered by Zhang et al. in 2005 in the rat stomach which is a main source of endogenous obestatin [264]. The name obestatin comes from the Latin word “obedere”, meaning devour, and “statin”, meaning suppression. This name was chosen due to the first results suggesting that it inhibits food intake. Obestatin is encoded by the same gene as ghrelin. Obestatin is composed of a 23-amino acid peptide derived from a 117-amino-acid preproghrelin by the posttranslational processing [264]. Its C-terminal amide group is thought to stabilize the peptide’s overall conformations [265]. The structure of human obestatin was characterized by α-helix followed by a single turn helix conformation between residues Pro^4^ and Gln^15^, and His^19^ and Ala^22^, respectively. These α-helical structures are critical for in vivo activity [266,267]. Obestatin, as well as ghrelin, are secreted mainly by the stomach [264]. Besides the stomach, the presence of obestatin is observed in other gastrointestinal organs. In rats, Zhao et al. have found the immunofluorescence staining for obestatin in the duodenum, jejunum, colon end endocrine pancreas [268]. Moreover, Dun et al. [269] have additionally observed obestatin immunoreactivity in rat myenteric plexus and in Leydig cells. A similar distribution of obestatin immunoreactive cells was observed in human tissues by Grönberg et al. [270], who have detected the presence of obestatin in the mucosa of the gastrointestinal tract, from cardia to ileum, in the pancreatic islets and epithelial cells in the ducts of mammary glands [270]. Moretti et al. [271] have explored obestatin localization in the male reproductive system. The immunoreactivity for obestatin has been found in Sertoli cells, the rete testis, efferent ductules, vas deferens, seminal vesicles, prostate and spermatozoa [271]. Volante et al. [272] have reported that, in fetal human tissue samples, obestatin is detected in the thyroid, pituitary, lung, pancreas and gastrointestinal tract. In human adult tissues, the obestatin protein expression is restricted to the pituitary, lung, pancreas and gastrointestinal tract. In contrast, in endocrine tumors, obestatin is expressed in a small fraction of thyroid, parathyroid, gastrointestinal and pancreatic neoplasms [272]. Alnema et al. [273] have examined the expression of obestatin by immunohistochemistry in oral biopsy specimens. Squamous cell carcinomas and benign tissue samples were positive for obestatin, and the expression of obestatin was decreased or absent in oral squamous cell carcinoma in relation to the cancer invasiveness [273].

Zhang et al. [264,274] and other authors [275,276,277] have proposed that obestatin exerts its biological effect by binding to the G protein-coupled receptor 39 (GPR39). However, numerous reports have questioned this concept [278,279,280], and some studies suggest that obestatin acts via the glucagon-like peptide 1 receptor (GLTP-1) [281,282,283]. However, the receptor for obestatin is still unknown.

Previous studies have reported that obestatin inhibits the motility of gastrointestinal tract [284,285], the secretion of vasopressin [286] and thirst [287], affecting the control of fluid homeostasis. Some studies indicate that obestatin stimulates exocrine pancreatic secretion [288].

In the central nervous system, obestatin inhibits anxiety and improves memory [289] and increases the deep non-rapidieye-movement (NREM) phase of sleep [290].

**Figure 3:**
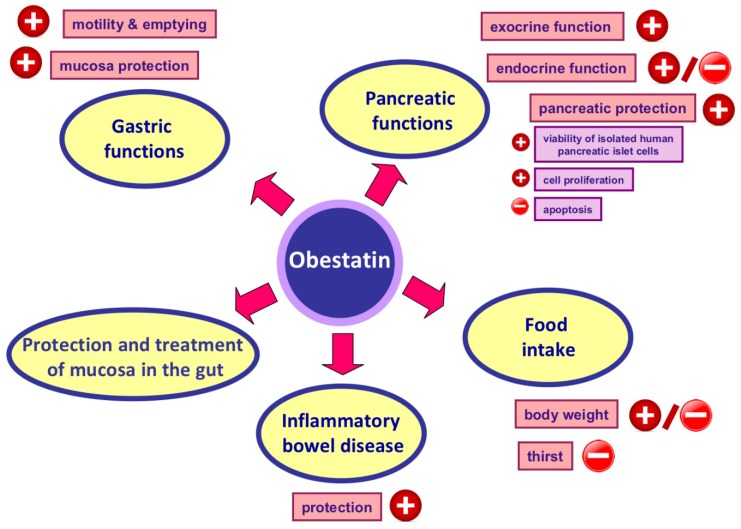
A schematic representation of obestatin in the gut. Plus means increases stimulates; minus meas inhibits.

Obestatin, like ghrelin, shows antioxidant and anti-inflammatory effects in many organs in the human body. Therefore, the effects of obestatin in the prevention and/or treatment of various diseases have been extensively investigated. The protective or therapeutic effect of obestatin has been observed in the heart [291,292,293], kidney [294] and skeletal muscle [295]. In the gut, animal studies have shown that obestatin accelerates the healing of chronic gastric ulcer evoked by acetic acid [296] (Figure 3), attenuates mesenteric ischemia/reperfusion-induced oxidative injury of the ileum and lung [297], inhibits the development of acetic acid-induced colitis [298], reduces the severity of experimental colitis evoked by trinitrobenzene sulfonic acid [299] and accelerates the healing of acetic acid-induced colitis in rats [300]. In the pancreas, obestatin promotes the survival of pancreatic beta-cells and human islets and induces the expression of genes involved in the regulation of beta-cell mass and function [282], as well as enhances the in vitro generation of pancreatic islets through the regulation of developmental pathways [301]. Moreover, obestatin has been shown to inhibit the development of acute pancreatitis evoked by cerulein [302] or pancreatic ischemia followed by reperfusion [303], as well as exhibits a therapeutic effect in these models of acute pancreatitis [304,305], leading to a reduction in the severity of acute pancreatitis and the acceleration of pancreatic recovery [302,303]. The protective and therapeutic effect was also shown in the experimental models of colitis. Previous studies have shown that pretreatment with obestatin inhibits the development of dextran sulfate sodium-induced colitis [226] and acetic acid-induced colitis in rats [298]. The therapeutic effect of obestatin has been found in experimental colitis evoked by acetic acid [300] or trinitrobenzene sulfonic acid [299]. The beneficial effect of obestatin has been also found in the liver, where it exhibits a protective effect against ischemia/reperfusion-induced hepatic injury [306], as well as inhibits and reverses the development of nonalcoholic fatty liver disease [307].

Mechanisms of protective and therapeutic effects of obestatin involve, among others, the improvement of blood flow in exposed to damaging factor organs, the amelioration of cell vitality and cell proliferation, a reduction of the expression and presence of pro-inflammatory IL1-β and TNF-α, a decrease in leukocytosis and tissue activity of myeloperoxidase [296,299], the limitation of malondialdehyde (MDA) tissue content and an increased SOD level [303]. Moreover, obestatin exhibits an antiapoptotic effect, and the mechanism of this effect involves the activation of PI3K, PKC-epsilon, PKC-delta and ERK1/2 signaling [291] and the activation of an NO/soluble guanylate cyclase (sGC)/PKG pathway [292].

## 8. Conclusions

Oral mucosistis is a common and severe side effect of anticancer therapy. The presence of oral lesions is responsible for the development of local and systemic detrimental effects such as severe oral pain, difficulties with the swallowing of solid and liquid foods (dysphagia), trouble with speaking (dysarthria), opioid use, dehydration and the introduction of tube feeding. Moreover, mucosal lesions may be a gateway for opportunistic infection and systemic inflammation. Oral mucositis and its complications are associated with morbidity and mortality, the lowering of the quality of life and the increase in the cost of treatment. There are numerous strategies for the prevention or treatment of oral mucositis; however, their effectiveness is limited and does not correspond to expectations. Ghrelin and obestatin exhibit anti-inflammatory, antioxidative and antiapoptotic effects. Numerous experimental studies have shown that ghrelin and obestatin exhibit a protective and healing-promoting effect in different models of organ damage, including mucosa in the gut. These findings suggest that ghrelin and/or obestatin may be useful in the prevention and treatment of oral mucositis. Hence, further animal and clinical studies should be performed to clarify their usefulness in this disease.
